# Fine particulate matter estimated by mathematical model and hospitalizations for pneumonia and asthma in children

**DOI:** 10.1016/j.rppede.2015.12.005

**Published:** 2016

**Authors:** Ana Cristina Gobbo César, Luiz Fernando Costa Nascimento, Katia Cristina Cota Mantovani, Luciana Cristina Pompeo Vieira

**Affiliations:** a Instituto Federal de Educação, Ciência e Tecnologia de São Paulo, Bragança Paulista, SP, Brazil; b Departamento de Energia, Universidade Estadual Paulista Júlio de Mesquita Filho, Guaratinguetá, SP, Brazil

**Keywords:** Asthma, Pneumonia, Air pollutants, Particulate matter, Child health, Mathematical models

## Abstract

**Objective:**

To estimate the association between exposure to fine particulate matter with an aerodynamic diameter <2.5 microns (PM_2.5_) and hospitalizations for pneumonia and asthma in children.

**Methods:**

An ecological study of time series was performed, with daily indicators of hospitalization for pneumonia and asthma in children up to 10 years of age, living in Taubaté (SP) and estimated concentrations of PM_2.5_, between August 2011 and July 2012. A generalized additive model of Poisson regression was used to estimate the relative risk, with lag zero up to five days after exposure; the single pollutant model was adjusted by the apparent temperature, as defined from the temperature and relative air humidity, seasonality and weekday.

**Results:**

The values of the relative risks for hospitalization for pneumonia and asthma were significant for lag 0 (RR=1.051, 95%CI; 1.016 to 1.088); lag 2 (RR=1.066, 95%CI: 1.023 to 1.113); lag 3 (RR=1.053, 95%CI: 1.015 to 1.092); lag 4 (RR=1.043, 95%CI: 1.004 to 1.088) and lag 5 (RR=1.061, 95%CI: 1.018 to 1.106). The increase of 5mcg/m^3^ in PM_2.5_ contributes to increase the relative risk for hospitalization from 20.3 to 38.4 percentage points; however, the reduction of 5µg/m^3^ in PM_2.5_ concentration results in 38 fewer hospital admissions.

**Conclusions:**

Exposure to PM_2.5_ was associated with hospitalizations for pneumonia and asthma in children younger than 10 years of age, showing the role of fine particulate matter in child health and providing subsidies for the implementation of preventive measures to decrease these outcomes.

## Introduction

Air pollution has been associated with increased risk of death, chronic diseases and particularly respiratory diseases in children.^1^^-^ 4 This finding may be explained by the respiratory system immaturity, as lung development is progressive and continuous until the age of 10,^5^ as well as due to the increased emission of pollutants into the atmosphere.^6^^,^ 7

A study carried out in 20 cities located in two different regions of California (USA), suggested that the level of exposure to fine particulate matter <2.5 microns of aerodynamic diameter (PM_2.5_) in children between 1 and 9 years is associated with increased number of hospitalizations caused by acute respiratory infections such as pneumonia and asthma.^8^

Data from the Ministry of Health reported more than 570,000 admissions in 2011 related to children up to 10 years of age, which generated costs of more than 400 million reais in Brazil. In the state of São Paulo, there were approximately 100,000 hospitalizations generating costs of about 85 million reais.^9^

Pneumonia and asthma are multifactorial diseases. The risk factors most commonly associated with their occurrence are low birth weight, the presence of smokers at home, lack of breastfeeding and exposure to air pollutants.^4^^,^ 7 ^,^ 10 ^,^ 11 Specifically in cases of asthma, vehicular traffic, particularly heavy vehicles, seems to be important.^3^

Among air pollutants, those generated by biomass burning, as well as by mobile sources involved with hospitalizations for respiratory diseases in children, we highlight the importance of PM_10_ and, among this matter, the fraction with PM_2.5_. This fraction, called fine particulate matter, has a diameter ranging between 0.1µm and 2.5µm (PM_2.5_) and represents between 60% and 70% of the total particulate matter.^12^ The use of logistic regression identified the association between exposure to PM_2.5_ and the increased risk of hospitalization for childhood bronchiolitis (OR=1.09; 95%CI: 1.04–1.14) in a study carried out in California.^13^

Fine particulate matter is directly emitted by the combustion of coal, oil, gas and wood. It is also secondarily formed from gaseous precursors. It primarily consists of sulphates, nitrates, chloride, ammonia compounds, elemental and organic carbon, as well as metals. It can remain in the atmosphere for long periods and travel long distances and reach the deepest portions of the respiratory tract.^14^

The particulate pollutants with larger diameters are retained in the upper airways, while those with smaller diameters reach the alveoli.^5^ In the lungs, these particles are phagocytosed by alveolar macrophages and removed through the action of cilia and the lymphatic system.^15^ However, the high levels of oxidants and pro-oxidants contained in the particulates can cause the formation of free radicals, trigger an inflammatory response with release of inflammatory mediators and cells and induce a subclinical inflammation of the respiratory system.^16^

In the case of PM_10_, its quantification is usually carried out through state environmental agencies monitoring stations; on the other hand, PM_2.5_ has few monitoring stations in the state of São Paulo (SP). However, studies using estimated concentrations of some air pollutants, among them PM_2.5_, provided by the mathematical model Chemical Coupled Aerosol and Tracer Transport Model to the Brazilian Developments on the Regional Atmospheric Modeling System (CCATT-BRAMS), have been used to evaluate the effects of air pollutant exposure.^17^^,^ 18 The use of estimated data becomes an option, in cases of cities where there are no monitoring stations for some or any pollutants.^11^^,^ 19

The objective of this study was to evaluate the role of PM_2.5_, estimated by a mathematical model, in hospitalizations for pneumonia and asthma in children up to 10 years of age, living in the city of Taubaté (SP), which has no monitoring station for this type of pollutant.

## Method

This was an ecological study of time series with daily indicators of hospitalization for respiratory diseases (International Classification of Diseases, ICD 10: J12.0–J18.9, J45.0, J45.1, J45.8, J45.9 and J46) in children up to 10 years of age and residents in Taubaté (SP), from August 1, 2011 to July 31, 2012. These data were obtained from the Department of Information and Informatics of the Unified Health System^9^ (DATASUS), in addition to the mean value of each admission for these diseases. We also calculated the estimated daily means of carbon monoxide (CO in ppb), nitrogen oxides (NO*x* in µg/m^3^) and fine particulate matter (PM_2.5_ in µg/m^3^), and a maximum of 8h for the ozone (O_3_ in µg/m^3^) obtained from the CCATT-BRAMS system.^17^

The temperature and humidity data were provided by the Weather Forecast and Climate Study Center of the National Institute of Space Research (Centro de Previsão do Tempo e Estudos Climáticos do Instituto Nacional de Pesquisas Espaciais – CPTEC-INPE) and, from these data, the apparent temperature was calculated, which is a function of temperature and humidity. The apparent temperature considers the physiological experience of combined exposure of humidity and temperature, and allows a more effective evaluation of the response of these variables on the individual's health.^20^

Variables related to apparent temperature, seasonality and calendar effects (day of the week) were included to fit the model.

Taubaté is located in Vale do Paraíba, 120km from the state capital, at coordinates 22°45′S and 45°30′W, with a population of 300,000 inhabitants in a territory of just over 625km^2^. It has an important industrial park with automotive and steel industries.^21^ The city is crossed by Via Dutra, a highway that connects the two largest cities in Brazil and has a fleet of approximately 200,000 automotive vehicles.^21^

Pearson's correlation coefficient test was used to evaluate the possible correlations between hospitalizations and the estimated levels of PM_2.5_. As the effects of exposure to environmental pollutants can lead to hospitalization on the same day or on subsequent days, the effects on the respiratory system were investigated on the day of admission (lag 0), and also on the subsequent 5 days (lags 1–5). The generalized additive model of Poisson regression was used because the outcome is a discrete quantitative variable. The hospitalization risk results refer to exposure to PM_2.5_ adjusted for apparent temperature, seasonality and weekday. The Statistica v7 program was used for the statistical analysis.

The estimated effects are the relative risks (RRs) corresponding to the increase of 5µg/m^3^ of PM_2.5_. To interpret the results, the RRs were converted into of percentage hospitalization risk increase (HRI). We applied the expression HRI=[exp(β*Δ*pol*)−1]*100, where *β* is the coefficient obtained from Poisson regression and Δ*pol* is the variation, in µg/m^3^, to be added in the concentration of the analyzed pollutant.^4^ This increase allows calculating the population-attributable fraction, that is, how many hospitalizations occurred as a result of this increase. The costs of hospitalization due to this increase in concentrations of the fine particulate matter were calculated. The 5% significance level was used in all analyses.

This study was approved by the Research Ethics Committee of Universidade de Taubaté (Opinion N. 068/12).

## Results

During the study period, there were 140 hospitalizations for pneumonia and asthma in children up to 10 years of age, living in Taubaté (SP). The mean number of daily admissions was 0.4±0.7, with a minimum of 0 and a maximum of 4. The estimated mean daily concentration of PM_2.5_ was 13.2±5.7µg/m^3^ and the minimum and maximum values were, respectively, 0.40–41.30µg/m^3^, with the values exceeding the limit of 25µg/m^3^ established by the State Decree N. 59113/13,^22^ on 8 days of the assessed period. The mean daily apparent temperature was 20.4±4.0°C. The descriptive analysis of the independent variables (pollutants and apparent temperature) is shown in Table 1.

**Table 1:** Descriptive analysis of air pollutants and apparent temperature (°C). Taubaté, São Paulo, 2011–2012.

	Mean (standard deviation)	Minimum–maximum
Carbon monoxide (ppb)	119.1 (39.3)	33.3–281.3
Ozone (µg/m^3^)	36.4 (16.2)	10.5–98.0
Nitrogen oxides (µg/m^3^)	2.1 (1.7)	0.2–15.8
Particulate matter <2.5 microns of aerodynamic diameter (µg/m^3^)	15.4 (3.8)	11.5–41.3
Apparent temperature	20.7 (3.8)	7.2–36.4

Table 2 shows the Pearson correlation between the study variables. The hospitalizations for pneumonia and asthma were positively correlated (*p*<0.05) with the PM_2.5_, seasonality and apparent temperature (*p*<0.01). The apparent temperature was positively correlated with the PM_2.5_ and seasonality (*p*<0.05). This justifies the use of such measures as adjusting variables in the model.

**Table 2:** Pearson's correlation matrix between the variables hospitalizations, particulate matter <2.5 microns of aerodynamic diameter, apparent temperature (°C), seasonality and weekday. Taubaté, São Paulo, 2011–2012.

Variables	Hospitalizations	Particulate matter <2.5 microns of aerodynamic diameter	Apparent temperature (°C)	Seasonality	Weekday
Hospitalizations	1				
Particulate matter <2.5 microns of aerodynamic diameter	0.21 b	1			
Apparent temperature (°C)	0.11 a	0.52 b	1		
Seasonality	0.28 b	0.34 b	0.32 b	1	
Weekday	−0.05	0.054	−0.04	−0.01	1

a Significant correlation for *p* <0.01.

b Significant correlation for *p* <0.05.

As for Poisson regression, the analysis with the single-pollutant model (PM_2.5_) adjusted by the apparent temperature, seasonality and weekday, Table 3 shows the coefficients and their respective standard errors. The effects of the pollutant on children's health were quantified by RR, which expresses the probability of an individual to develop a disease related to exposure to a risk factor.

**Table 3:** Values of the coefficients and standard errors for the exposure to particulate matter <2.5 microns of aerodynamic diameter, single-pollutant model adjusted for apparent temperature, seasonality and weekday, according to lags (lag) 0–5 days. Taubaté, São Paulo, 2011–2012.

Lag (days)	Coefficients (standard error)
*Lag 0*	**0.04982 (0.01755)**
*Lag 1*	0.03527 (0.02129)
*Lag 2*	**0.06449 (0.02154)**
*Lag 3*	**0.05177 (0.01871)**
*Lag 4*	**0.04434 (0.02056)**
*Lag 5*	**0.05940 (0.02100)**

The values in bold indicate *p* <0.05.

**Figure 1:**
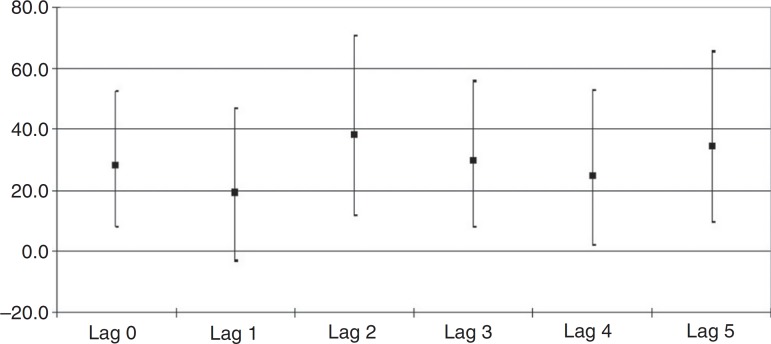
Percentage increases of the relative risks and corresponding 95% confidence intervals for all lags after an increment of 5µg/m^3^ in PM_2.5_ concentrations. Taubaté, Brazil, 2011–2012.

The RR and their respective confidence intervals (CIs), which were significant, according to the exposure to PM_2.5_ and hospitalization, refer to lag 0 (RR=1.051; 95%CI 1.016–1.088); lag 2 (RR=1.066; 95%CI 1.023–1.113); lag 3 (RR=1.053; 95%CI 1.015–1.092); lag 4 (RR=1.043; 95%CI 1.004–1.088) and lag 5 (RR=1.061; 95%CI 1.018–1.106). The RRs and corresponding 95%CIs for all lags are shown in Fig. 1.

**Figure 2:**
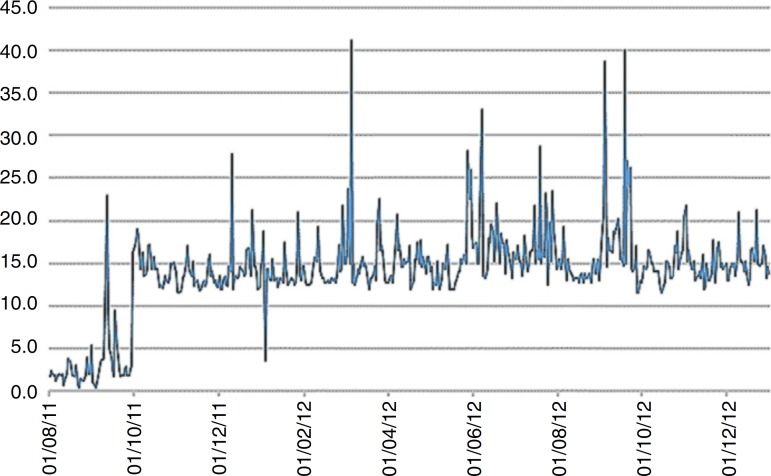
Daily values, in µg/m^3^, of the particulate matter <2.5 microns of aerodynamic diameter pollutant concentrations. Taubaté, Brazil, 2011–2012.

The increase in RRs with the increment of 5µg/m^3^ in PM_2.5_ concentrations imply in increases between 20.3 and 38.4 percentage points in hospitalization risks. Fig. 2 shows the daily PM_2.5_ concentrations during the study period.

## Discussion

This study used data on pollutants estimated by the CCATT-BRAMS system, which considers the atmospheric dynamics and demonstrated a positive association between exposure to PM_2.5_ and hospitalizations for pneumonia and asthma in children living in the city of Taubaté (SP).

The CCATT-BRAMS system is a mathematical model that comprehends South America and considers the emission and transport of different gases and aerosol particles, obtained by satellites, and generates daily estimates for different pollutants.^17^ The model provides PM_2.5_ measurements every 3h over a grid of cells with 30km×30km. Daily arithmetic means of PM_2.5_ concentrations were calculated based on the means of the set of cells that comprise the territory area of the assessed municipality.

The use of data estimated by this system, which has been validated by Ignotti et al.,^10^ Cesar et al.^11^ and Silva et al.,^19^ is advantageous when applied to cities where there are no pollution monitoring stations, as is the case of Taubaté. This allows the decrease in research costs and optimizes the process of analyzing the effects of air pollution on health. However, the fact that CCATT-BRAMS measures are estimated at an altitude of 40m, and not in regions closer to the ground, where the concentrations can be different, may represent a limitation of this study.^11^

During the study period, it was observed that hospitalizations for pneumonia and asthma in children aged up to 10 years showed a significant positive association with PM_2.5_, both on the exposure day (lag 0) and from the second to the fifth day of the exposure (lags 2–5), suggesting an acute effect to exposure. The acute effects manifest after a short time between exposure and the effects, which can be hours or days.^2^

Epidemiological studies show that exposure to gaseous pollutants and particulate matter is associated with a higher incidence of lower respiratory symptoms such as cough, dyspnea and wheezing, especially in children.^2^

In Taubaté (SP), the main source of air pollutants is the burning of fossil fuels by the vehicle fleet of the municipality, which has the highest number of vehicles per inhabitant (64.5 vehicles/100 inhabitants)^21^ when compared to the other cities located in Vale do Paraíba (SP). Another important contribution is brought on by the fleet that goes through Presidente Dutra interstate highway, consisting mainly of buses and heavy trucks. The study of recent data from the city of São José dos Campos (SP) showed that there was a decrease in hospitalizations for pneumonia in children with the reduction of SO_2_ and PM_10_ concentrations, but there was an association between exposure to carbon monoxide (CO) and ozone (O_3_) and hospitalizations. This suggests that the effects of control measures implemented in the period were neutralized by the increase in the vehicle fleet.^23^

In urban areas, exposure to air pollutants is characterized by long periods and low levels of pollutants,^3^ unlike air pollution originating from wildfires, of which main characteristic is the well-defined seasonality and high levels mainly of particulate matter.^19^ The burning of biomass, as shown by the results of Piracicaba (SP) and the Amazon region, has an important role in pollutant concentrations.^7^^,^ 10 ^,^ 11

Studies in Brazil show the influence of PM_10_ in the genesis of hospitalizations for respiratory diseases in children.^3^^,^ 4 ^,^ 24 However, the fine (PM_2.5_) particulate is scarcely studied, as it is not quantified by the environmental agency stations; recently, national studies were published demonstrating the role of this fraction in the genesis of hospital admissions for respiratory diseases.^10^^,^ 11 ^,^ 19 In Taubaté, the RRs of hospitalizations for pneumonia and asthma ranged between 4.1% and 6.3%. Although low, the risks were higher than those found in Piracicaba (SP).^11^ In Taubaté, an increase of 5µg/m^3^ in PM_2.5_ concentrations implies in significant increases in the risk of hospitalization, between 20.3 and 38.4 percentage points.

The findings of this study agree with those of other national articles^7^^,^ 10 ^,^ 11 and the results of Hertz-Picciotto et al.,^25^ who pointed out the role of this pollutant in hospitalizations for lower respiratory tract infections in preschool children in the Czech Republic. Karr et al.,^13^ using conditional logistic regression, showed that exposure to PM_2.5_ was associated with increased risk of hospitalization for childhood bronchiolitis (OR=1.09; 95%CI: 1.04–1.14). The aforementioned national studies used data estimated by models and the studies by Hertz-Picciotto et al.^25^ and Karr et al.^13^ used data collected by monitoring stations. The potential mechanisms by which PM_2.5_ can lead to respiratory diseases are numerous, including oxidative stress, structural damage, efficient transport of pathogens and immune system alterations, possibly correlated with carbon organic compounds.^26^

Another important point to be highlighted is the increased hospital costs due to hospitalizations for respiratory diseases associated with exposure to fine particulate matter.^27^ In this study, it was estimated that the reduction in 5µg/m^3^ in PM_2.5_ concentration in the atmosphere could contribute to the decrease in 38 pediatric hospitalizations for pneumonia and asthma in Taubaté, within the period of one year, which would result in savings of R$ 84,000 for the Brazilian Unified Health System (SUS).

This study has limitations, some of which are inherent to ecological studies; the fact of working with secondary data, even those from official sources commonly used for studies on this subject, allows the possibility of diagnosis coding errors, in addition to the fact that these data do not provide information about comorbidities, smoking in the home (passive smoking) and parental education, among other associated factors. This source has the basic accounting purpose and the provided data on hospital admissions are restricted to the users of the Unified Health System, excluding information on children treated through health insurance companies or in private clinics. Another limitation of this study may lie in the fact the CCATT-BRAMS measures are estimated at an altitude of 40m, and not in areas closer to the ground, where concentrations can be different; furthermore, it was assumed that the concentrations were homogeneous and that children were equally exposed to air pollutants, especially to the fine particulate, considerations that could represent another possible limitation.

Despite the strong biological plausibility, our results cannot allow us to presume that there are causal associations. Further investigations are needed on the roles of other pollutants, such as O_3_, PM_10_, CO, NO_2_ and metals, which have been associated with a diversity of respiratory diagnoses.

Therefore, exposure to PM_2.5_ was associated with hospitalizations for pneumonia and asthma in children younger than 10 years of age in a medium-sized city. This information corroborates the findings from other studies and demonstrates the important role of fine particulate in children's health, in addition to providing subsidies for the implementation of preventive measures to reduce these outcomes.
