# Supplementary material for: Fine particulate matter estimated by mathematical model and hospitalizations for pneumonia and asthma in children
Source: Rev Paul Pediatr. 2016 Jan-Mar;34(1):18–23. doi: 10.1016/j.rppede.2015.12.005 (PMC4795717; doi:10.1016/j.rppede.2015.12.005)
Supplement: Translation

# Translation: Material particulado fino estimado por modelo matemático e internações por pneumonia e asma em crianças

**Authors:** Ana Cristina Gobbo César, Luiz Fernando Costa Nascimento, Katia Cristina Cota Mantovani, Luciana Cristina Pompeo Vieira

**Affiliations:** a Instituto Federal de Educação, Ciência e Tecnologia de São Paulo, Bragança Paulista, SP, Brasil; b Departamento de Energia, Universidade Estadual Paulista Júlio de Mesquita Filho, Guaratinguetá, SP, Brasil

**Keywords:** Asma, Pneumonia, Poluentes do ar, Material particulado, Saúde da criança, Modelos matemáticos

## Abstract

**Objetivo:**

Estimar a associação entre exposição ao material particulado fino com diâmetro aerodinâmico inferior a 2,5 micra (PM_2.5_) e as internações por pneumonia e asma em crianças.

**Métodos:**

Estudo ecológico de séries temporais com indicadores diários de internação por pneumonia e asma, em crianças com até 10 anos, residentes em Taubaté (SP), e concentrações estimadas de PM_2.5_, entre agosto de 2011 e julho de 2012. Modelo aditivo generalizado de regressão de Poisson foi usado para estimar o risco relativo, com defasagem de zero até cinco dias após a exposição; o modelo unipoluente foi ajustado pela temperatura aparente, medida definida a partir da temperatura e umidade relativa do ar, sazonalidade e dia da semana.

**Resultados:**

Os valores dos riscos relativos para internações por pneumonia e asma foram significativos para lag 0 (RR=1,051; IC95% 1,016-1,088); lag 2 (RR=1,066; IC95% 1,023-1,113); lag 3 (RR=1,053; IC95% 1,015-1,092); lag 4 (RR=1,043; IC95% 1,004-1,088) e no lag 5 (RR=1,061; IC95% 1,018-1,106). O incremento de 5mcg/m^3^ de PM_2.5_ contribui para aumento no risco relativo para internações entre 20,3 a 38,4 pontos percentuais; no entanto, a diminuição de 5µg/m^3^ na concentração do PM_2.5_ resulta em menos 38 internações.

**Conclusões:**

A exposição ao PM_2.5_ esteve associada às internações por pneumonia e asma em crianças menores de 10 anos, mostrou o papel do material particulado fino na saúde da criança e forneceu subsídios para implantação de medidas preventivas para diminuírem esses desfechos.

## Introdução

A poluição do ar tem sido associada ao aumento no risco de morte, doenças crônicas e, principalmente, doenças respiratórias em crianças.^1^^-^ 4 Tal achado pode ser explicado pela imaturidade do sistema respiratório, pois o desenvolvimento dos pulmões é progressivo e contínuo até os 10 anos,^5^ assim como pelo aumento da emissão de poluentes na atmosfera.^6^^,^ 7

Estudo feito em 20 cidades localizadas em duas diferentes regiões da Califórnia (EUA) sugeriu que o nível de exposição ao material particulado fino com menos de 2,5 micra de diâmetro aerodinâmico (PM_2.5_) em crianças entre um e nove anos está associado ao aumento de hospitalizações causadas por infecções respiratórias aguda, como a pneumonia e a asma.^8^

Dados do Ministério da Saúde relatam mais de 570 mil internações em 2011 referentes a crianças com até 10 anos, que geraram uma despesa de mais de 400 milhões de reais no Brasil. No estado de São Paulo foram cerca de 100 mil internações com despesa de cerca de 85 milhões de reais.^9^

A pneumonia e a asma são doenças de caráter multifatorial. Os fatores de risco mais comumente associados à sua ocorrência são o baixo peso ao nascer, a presença de fumantes na residência, a falta de aleitamento materno e a exposição a poluentes do ar.^4^^,^ 7 ^,^ 10 ^,^ 11 Especificamente nos casos de asma, o tráfego veicular, particularmente de veículos pesados, parece ser importante.^3^

Dentre os poluentes do ar, tanto os gerados por queima de biomassa como os gerados por fontes móveis, implicados com as internações por doenças respiratórias em crianças, se destaca o material particulado com menos de 10 micra de diâmetro aerodinâmico e, dentre esse material, a fração com menos de 2,5 micra de diâmetro aerodinâmico. Essa fração, chamada de particulado fino, tem um diâmetro que varia entre 0,1µm e 2,5µm (PM_2.5_) e representa entre 60% e 70% do total do material particulado.^12^ Com o uso da regressão logística identificou-se a associação entre a exposição ao PM_2.5_ e o aumento do risco de internação por bronquiolite infantil (OR=1,09; IC95% 1,04-1,14) em um estudo feito na Califórnia.^13^

O material particulado fino é diretamente emitido pela combustão de carvão, óleo, gasolina e madeira. É também formado secundariamente de precursores gasosos. É composto principalmente por sulfatos, nitratos, cloreto, compostos de amônia e carbono orgânico e elementar além de metais. Pode permanecer muito tempo na atmosfera e percorrer longas distâncias e atingir porções mais profundas do trato respiratório.^14^

Os poluentes particulados com maior diâmetro são retidos nas vias aéreas superiores, enquanto os de menor diâmetro chegam até os alvéolos.^5^ Nos pulmões, essas partículas são fagocitadas pelos macrófagos alveolares e removidas pela ação dos cílios e do sistema linfático.^15^ Entretanto, as altas concentrações de oxidantes e pró-oxidantes contidos nos materiais particulados podem provocar a formação de radicais livres, iniciar uma resposta inflamatória com a liberação de células e mediadores inflamatórios, provocar uma inflamação subclínica do sistema respiratório.^16^

No caso do PM_10_, sua quantificação costuma ser feita por estações de monitoramento de agências ambientais estaduais; por outro lado, o PM_2.5_ conta com poucas estações medidoras no Estado de São Paulo. Entretanto, estudos que usaram concentrações estimadas de alguns poluentes do ar, dentre eles, o PM_2.5_, disponibilizadas pelo modelo matemático Chemical Coupled Aerosol and Tracer Transport Model to the Brazilian Developments on the Regional Atmospheric Modeling System (CCATT-BRAMS) têm sido usados na avaliação dos efeitos da exposição aos poluentes do ar.^17^^,^ 18 O uso de dados estimados torna-se uma opção, no caso de cidades onde não há estações medidoras de certos ou quaisquer poluentes.^11^^,^ 19

O objetivo deste estudo foi avaliar o papel do PM_2.5_ estimado por modelo matemático, nas internações por pneumonia e asma em crianças com até 10 anos, residentes em Taubaté (SP), que não tem estação medidora desse tipo de poluente.

## Método

Estudo ecológico de séries temporais com indicadores diários de internação por doenças respiratórias (CID 10: J12.0 a J18.9; J45.0; J45.1; J45.8; J45.9 e J46), em crianças com até 10 anos e residentes em Taubaté (SP), de 1° de agosto de 2011 a 31 de julho de 2012. Esses dados foram obtidos do Departamento de Informações e Informática do Sistema Único de Saúde^9^ (Datasus), além do valor médio de cada internação por essas doenças. Também foram calculadas as médias diárias estimadas de monóxido de carbono (CO em ppb), óxidos de nitrogênio (NOx em µg/m^3^) e material particulado fino (PM_2.5_ em µg/m^3^) e o máximo de oito horas para o ozônio (O_3_ em µg/m^3^) obtidos do sistema CCATT-BRAMS.^17^

Os dados de temperatura e umidade foram fornecidos pelo Centro de Previsão do Tempo e Estudos Climáticos do Instituto Nacional de Pesquisas Espaciais (Cptec-Inpe) e, a partir deles, foi calculada a temperatura aparente, que é função da temperatura e umidade. A temperatura aparente considera a experiência fisiológica da exposição combinada da umidade e temperatura e permite avaliar com maior eficiência a resposta dessas variáveis sobre a saúde do indivíduo.^20^

Variáveis relacionadas a temperatura aparente, sazonalidade e efeitos de calendário (dia da semana) foram incluídas para ajustar o modelo.

Taubaté fica no Vale do Paraíba paulista, a 120 km da capital do estado, nas coordenadas 22° 45′ S e 45° 30′ W, com uma população de 300 mil habitantes, num território com pouco mais de 625 km^2^. Conta com um importante parque industrial com indústrias automobilística e siderúrgica.^21^ É cortada pela Via Dutra, que liga as duas maiores cidades do Brasil e recebe uma frota de aproximadamente 200 mil veículos.^21^

Para avaliar as possíveis correlações entre as internações e os níveis estimados de PM_2.5_ usou-se o teste coeficiente de correlação de Pearson. Como os efeitos da exposição aos poluentes ambientais podem acarretar internação no mesmo dia ou em dias posteriores, foram investigados os efeitos no aparelho respiratório no dia da internação (*lag* 0) e também nos cinco dias subsequentes (*lag* 1 a *lag* 5). Usou-se o modelo aditivo generalizado de regressão de Poisson, pois o desfecho é uma variável quantitativa discreta. Os resultados do risco de internação se referem à exposição ao PM_2.5_ ajustado por temperatura aparente, sazonalidade e dia da semana. Usou-se o programa Statistica v7 para as análises.

Os efeitos estimados são os riscos relativos (RR), correspondentes ao incremento de 5 µg/m^3^ de PM_2.5_. Para interpretar os resultados, os RR foram convertidos para aumentos percentuais do risco de internação (ARI). Foi aplicada a expressão ARI = [exp (β*Δpol)-1]*100, βo coeficiente obtido da regressão de Poisson e Δpol a variação, em µg/m^3^, a ser acrescentada na concentração do poluente em análise.^4^ Esse aumento permite calcular a fração atribuível populacional, isto é, quantas internações ocorreram em decorrência desse aumento. Foram calculados os gastos com internações em função desse aumento nas concentrações do material particulado fino. O nível de significância de 5% foi adotado em todas as análises.

O presente trabalho foi aprovado pelo Comitê de Ética em Pesquisa da Universidade de Taubaté (Parecer n° 068/12).

## Resultados

No período avaliado, ocorreram 140 internações por pneumonia e asma em crianças até 10 anos, residentes em Taubaté (SP). A média diária de internações foi de 0,4±0,7, com um mínimo de zero e um máximo de quatro. A concentração média diária estimada de PM_2.5_ foi de 13,2±5,7µg/m^3^ e os valores mínimo e máximo foram, respectivamente, 0,40-41,30µg/m^3^; ultrapassaram o limite de 25µg/m^3^ estabelecido pelo Decreto Estadual n° 59113/13,^22^ em oito dias do período avaliado. A média diária da temperatura aparente foi 20,4±4,0 °C. A análise descritiva das variáveis independentes (poluentes e temperatura aparente) encontra-se na tabela 1.

**Tabela 1:** Análise descritiva dos poluentes do ar e temperatura aparente (TA°C). Taubaté, SP, 2011‐2012

	Média (dp)	Mínimo‐máximo
CO (ppb)	119,1 (39,3)	33,3–281,3
O_3_ (µg/m^3^)	36,4 (16,2)	10,5–98,0
Nox (µg/m^3^)	2,1 (1,7)	0,2–15,8
PM_2,5_ (µg/m^3^)	15,4 (3,8)	11,5–41,3
TA	20,7 (3,8)	7,2–36,4

A tabela 2 apresenta a correlação de Pearson entre as variáveis do estudo. As internações por pneumonia e asma apresentaram correlação positiva (*p*<0,05) com o PM_2.5_, sazonalidade e temperatura aparente (*p*<0,01). A temperatura aparente apresentou correlação positiva com o material particulado e sazonalidade (*p*<0,05). Isso justifica o uso de tais medidas como variáveis de ajuste no modelo.

**Tabela 2:** Matriz de correlação de Pearson entre as variáveis internações, PM_2.5_, temperatura aparente (TA°C), sazonalidade (SZ) e dia da semana (DS). Taubaté, SP, 2011‐2012

Variáveis	Internações	PM_2.5_	TA°C	SZ	DS
Internações	1				
PM_2.5_	0,21 b	1			
TA°C	0,11 a	0,52 b	1		
SZ	0,28 b	0,34 b	0,32 b	1	
DS	−0,05	0,054	−0,04	−0,01	1

a Correlação significativa para *p* <0,01.

b Correlação significativa para *p* <0,05.

Quanto à regressão de Poisson, a análise com o modelo unipoluente (PM_2.5_) ajustado pela temperatura aparente, sazonalidade e dia da semana, os coeficientes e respectivos erros padrão obtidos estão apresentados na tabela 3. Os efeitos do poluente sobre a saúde das crianças foram quantificados por meio do risco relativo (RR), que expressa a probabilidade de um indivíduo desenvolver uma doença relativa à exposição a um fator de risco.

**Tabela 3:** Valores dos coeficientes e respectivos erros padrão para a exposição ao PM_2.5_, modelo unipoluente ajustado pela temperatura aparente, sazonalidade e dia da semana, segundo defasagens ( *lag* ) de 0 a 5 dias. Taubaté, SP, 2011‐2012

Defasagem (dias)	Coeficientes (erro padrão)
*Lag 0*	**0,04982 (0,01755)**
*Lag 1*	0,03527 (0,02129)
*Lag 2*	**0,06449 (0,02154)**
*Lag 3*	**0,05177 (0,01871)**
*Lag 4*	**0,04434 (0,02056)**
*Lag 5*	**0,05940 (0,02100)**

Os valores em negrito apontam *p* <0,05.

Os RR e respectivos intervalos de confiança de 95% que se mostraram significativos, segundo a exposição ao PM_2.5_ e internação, se referem ao *lag* 0 (RR=1,051; IC95% 1,016-1,088); *lag* 2 (RR=1,066; IC95% 1,023-1,113); *lag* 3 (RR=1,053; IC95% 1,015-1,092); *lag* 4 (RR=1,043; IC95% 1,004-1,088) e *lag* 5 (RR=1,061; IC95% 1,018-1,106). Os riscos relativos e correspondentes intervalos de confiança de 95% para todos os *lags* estão apresentados na figura 1.

**Figura 1:** Aumentos percentuais dos riscos relativos e correspondentes intervalos de confiança de 95% para todos os *lags* após incremento de 5µg/m^3^ nas concentrações de PM_2.5_. Taubaté, SP, 2011-2012.

Os aumentos nos riscos relativos com o incremento de 5µg/m^3^ nas concentrações de PM_2.5_ implicam aumentos entre 20,3 a 38,4 pontos percentuais nos riscos de internação. Na figura 2 são observadas as concentrações diárias do PM_2.5_ durante o período de estudo.

**Figura 2:** Valores diários, em µg/m^3^, das concentrações do poluente PM_2.5_. Taubaté, SP, 2011-2012.

## Discussão

Este estudo usou dados de poluentes estimados pelo sistema CCATT-BRAMS, o qual considera a dinâmica atmosférica, e demonstrou a associação positiva entre a exposição ao PM_2.5_ e as internações por pneumonia e asma em crianças residentes em Taubaté (SP).

O sistema CCATT-BRAMS é um modelo matemático que abrange a América do Sul e considera a emissão e o transporte de diferentes gases e partículas de aerossóis, obtidos por satélites, e gera estimativas diárias para diferentes poluentes.^17^ O modelo fornece medições de PM_2.5_ a cada três horas sobre uma grade de células com 30×30 km^2^. Médias aritméticas diárias de concentrações do PM_2.5_ foram calculadas com base nas médias do conjunto de células que compõem a área territorial do município sob investigação.

O uso dos dados estimados por esse sistema, validados por Ignotti *et al*.,^10^ Cesar *et al*.^11^ e Silva *et al*.,^19^ é vantajoso ao ser aplicado em cidades em que não há estações medidoras de poluentes, como é o caso de Taubaté. Isso permite baratear os custos de pesquisa e agilizar o processo de análise dos efeitos da poluição atmosférica na saúde. Contudo, o fato de as medidas do CCATT-BRAMS serem estimadas numa altitude de 40m, e não em regiões mais próximas do solo, em que as concentrações podem ser diferentes, pode representar uma limitação deste estudo.^11^

Durante o período de estudo, observou-se que as internações por pneumonia e asma em crianças até 10 anos apresentaram associação positiva e significativa com o PM_2.5_, tanto no dia da exposição (*lag* 0) quanto do segundo até o quinto dia da exposição (*lag* 2 a 5), o que sugere uma ação aguda à exposição. Os efeitos agudos se manifestam após um curto tempo entre a exposição e os efeitos, que pode ser de horas ou dias.^2^

Estudos epidemiológicos evidenciam que a exposição a poluentes gasosos e material particulado está associada a maior incidência de sintomas das vias aéreas inferiores, como tosse, dispneia e sibilância, especialmente em crianças.^2^

Em Taubaté (SP), a fonte principal dos poluentes é a queima de combustíveis fósseis pela frota veicular do município que apresenta o maior número de veículos por habitantes (64,5 veículos/100 habitantes)^21^ em comparação com as demais cidades localizadas no Vale do Paraíba (SP). Outra importante contribuição é dada pela frota que trafega pela rodovia interestadual Presidente Dutra, composta principalmente por ônibus e veículos pesados de carga. Estudo feito com dados recentes de São José dos Campos (SP) mostrou que houve uma diminuição das internações por pneumonias em crianças com a diminuição das concentrações de SO_2_ e PM_10_, mas houve associação entre exposição ao monóxido de carbono (CO) e ozônio (O_3_) e as internações. Isso sugere que os efeitos de medidas de controle adotadas no período foram neutralizados pelo aumento da frota de veículos.^23^

Nos centros urbanos, a exposição aos poluentes atmosféricos é caracterizada por longos períodos e baixos níveis de poluentes,^3^ diferentemente da poluição do ar originária de queimadas, que tem como principal característica a sazonalidade bem definida e elevados níveis principalmente de material particulado.^19^ A queima de biomassa, como mostram os resultados de Piracicaba (SP) e da região amazônica, tem o papel relevante nas concentrações dos poluentes.^7^^,^ 10 ^,^ 11

Estudos feitos no Brasil mostram a influência do material particulado com menos de 10 micra de diâmetro aerodinâmico (PM_10_) na gênese de internações por doenças respiratórias em crianças.^3^^,^ 4 ^,^ 24 No entanto, o particulado fino PM_2.5_ é pouco estudado pelo fato de as estações das agências ambientais não o quantificarem; recentemente, foram publicados artigos nacionais que mostraram o papel dessa fração na gênese das internações por doenças respiratórias.^10^^,^ 11 ^,^ 19 Em Taubaté, os riscos relativos para internações por pneumonias e asma variaram entre 4,1% e 6,3%. Mesmo de pequena monta, foram maiores do que os encontrados em Piracicaba (SP).^11^ Em Taubaté, o incremento de 5 µg/m^3^ nas concentrações do PM_2.5_ implica aumentos consideráveis no risco de internação, entre 20,3 e 38,4 pontos percentuais.

Os achados deste estudo concordam com os de outros artigos nacionais^7^^,^ 10 ^,^ 11 e com os resultados de Hertz-Picciotto *et al*.,^25^ que apontaram o papel desse poluente nas internações por doenças do trato respiratório inferior em pré-escolares da República Checa. Karr *et al*.,^13^ com o uso da regressão logística condicional, mostraram que exposições ao PM_2.5_ estiveram associadas ao aumento do risco para hospitalização por bronquiolite infantil (OR=1,09; IC95%: 1,04-1,14). Os estudos nacionais citados usaram dados estimados por modelos e os de Hertz-Picciotto *et al*.^25^ e de Karr *et al*.^13^ usaram dados coletados por estações medidoras. Os mecanismos potenciais pelos quais o PM_2.5_ pode levar a doenças respiratórias são numerosos, incluindo estresse oxidativo, dano estrutural, transporte eficiente de micróbios patogênicos e alteração no sistema imunológico, possivelmente correlacionados com compostos orgânicos com carbono.^26^

Outro ponto importante a ser destacado é o aumento dos custos hospitalares pelas internações por doenças respiratórias associadas à exposição ao material particulado fino.^27^ Neste estudo, estimou-se que a diminuição em 5µg/m^3^ na concentração de PM_2.5_ na atmosfera poderia contribuir para a redução em 38 internações pediátricas por pneumonia e asma de Taubaté, nesse período de um ano, e gerar a economia de R$ 84 mil para o Sistema Único de Saúde (SUS).

Este estudo apresenta limitações, algumas próprias dos estudos ecológicos; o fato de se trabalhar com dados secundários, mesmo de fonte oficial comumente usada para estudos sobre esse tema, permite supor erros na codificação dos diagnósticos, além de esses dados não informarem sobre comorbidades, tabagismo na residência (fumante passivo) e escolaridade parental, entre outros fatores associados. Essa fonte tem a finalidade básica contábil e os dados fornecidos sobre as internações se restringem aos usuários do Sistema Único de Saúde, excluem informações sobre crianças atendidas por planos de saúde ou de forma particular. Outra limitação deste estudo pode residir no fato de as medidas do CCATT-BRAMS serem estimadas numa altitude de 40m, e não em regiões mais próximas do solo, onde as concentrações podem ser diferentes; além disso, assumiu-se que as concentrações eram homogêneas e que as crianças se expuseram igualmente aos poluentes do ar, em especial ao particulado fino, considerações que podem representar outra possível limitação.

Apesar da forte plausibilidade biológica, os nossos resultados não permitem presumir que existam associações causais. São necessárias investigações mais aprofundadas dos papéis de outros poluentes, como O_3_, PM_10_, CO, NO_2_ e metais, que têm sido associados com uma variedade de diagnósticos respiratórios.

Dessa forma, a exposição ao material particulado com menos de 2,5 micra de diâmetro aerodinâmico esteve associada às internações por pneumonia e asma em crianças menores de 10 anos, em uma cidade de porte médio. Essas informações corroboram os achados em outros estudos e mostram o papel importante do particulado fino na saúde da criança, além de fornecer subsídios para implantação de medidas preventivas para diminuir esses desfechos.
